# Health benefits of wolfberry (Gou Qi Zi, *Fructus barbarum* L.) on the basis of ancient Chineseherbalism and Western modern medicine

**Published:** 2021

**Authors:** Sun Wenli, Mohamad Hesam Shahrajabian, Cheng Qi

**Affiliations:** 1 *Biotechnology Research Institute, Chinese Academy of Agricultural Sciences, Beijing 100081, China*; 2 *Nitrogen Fixation Laboratory, Qi Institute, Building C4, No. 555, Chuangye Road, Jiaxing 314000, Zhejiang, China*; 3 *Hebei Agricultural University, College of Life Sciences, Baoding, Hebei, 071000, China; Global Alliance HeBAU-CLS&HeQiS for BioAl-Manufacturing, Baoding, Hebei 071000, China*; † * Equal first author*

**Keywords:** Health beneficial, Wolfberry, Chinese herbalism, Western medicine

## Abstract

**Objective:**

Goji berry has been used for thousand years inTraditional Chinese Medicine (TCM) in China and other Asian countries as foods to promote health and as drugs to treat diseases. It has been claimed this important medicinal crop is the good source of compounds with valuable nutritional and bioactive properties which can also provide industrial sustainability in organic life.

**Materials and Methods:**

All relevant papers in English language were collected. The keywords of wolfberry, goji berry, Chinese herbalism and western Medicine were searched in Google Scholar, Scopus, Research Gate and PubMed.

**Results:**

Besides its uses in food and culinary, wolfberry has long played important roles in TCM where they are believed to enhance immune system function, improve eyesight, protect liver, boost sperm production and improve circulation, among other effects. TCM calls for wolfberry to be prepared as a decoction or ground into a powder and mixed with other herbs. Additionally, Gojiberry is rich in ascorbic acid, thiamine and riboflavin. Moreover, Gojiberrycontains carbohydrates, organic acids, and so many minerals like potassium, sodium, phosphorus, magnesium, iron, calcium, zinc and selenium.

**Conclusion:**

This review article allowed verifying that wolfberry as asource of compounds with valuable nutritional and bioactive properties.

## Introduction

Traditional Chinese Medicine (TCM) originatedin ancient China and it hasa 5000-year history. Rooted in ancient Eastern philosophies such as Taoism, TCM focuses on a holistic view between humans and nature (Shahrajabian et al., 2018 ▶, Shahrajabian et al., 2019 ▶). About 5000 traditional remedies are available in China; they account for approximately one fifth of the entire Chinese pharmaceutical market.In TCM, it is believed that food and medicine come from the same origin but with different uses and applications (Shahrajabian et al., 2020a ▶, b, c, d.) Cultivation of Chinese medicinal herbs and usage of TCM significantly help to promote sustainable agricultural development via growing demand for organic and herbal products in different regions. Chinese medicinal plants, both endemic and widespread, must be preserved since these plants are renewable sources of new drugs. On the basis of economic prospects, organic farming of goji berry may lead to increase more market opportunity, to maintain high market price, to achieve optimal quality and economic returns and to secure economic growth and social stability. In these years, since TCM has become more integrated into medical practice in the West, there is a need to bridge conceptual and practical differences between Western medicine and Chinese medicine. Goji berry (*L. barbarum*) is known as an extremely nutritious food in Asia, and it has been extensively eaten raw, consumed as juice or wine, brewed into herbal tea or prepared as a tincture, eaten as salads and used widely in other culinary preparations. Its leaves are prepared as a tea. Besides, its uses in food and culinary, goji berry has important roles in TCM. The aim of this review is to survey composition, potential health benefits and pharmacological benefits of goji berry.

## Materials and Methods

Literature search was conducted in MEDLINE, RESEARCH GATE, SCOPUS, PUBMED and Google scholar databases. The keywords were wolfberry, goji berry, Chinese herbalism and western Medicine. 

## Results

**Wolfberry occurrence, cultivation, chemical constituents, and nutrient and chemical composition and its role in TCM**

**Figure 1:**
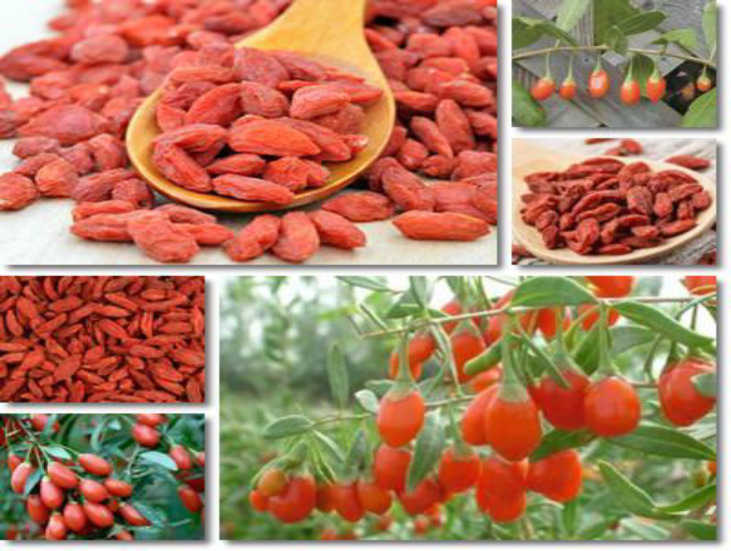
Fresh and dried gojiberry (Photo courtesy: The authors of the present article)

Goji (*L. Barbarum* L.) which is also called wolfberry is very hard, spiny, shrubby vines in the tomato-nightshade family Solanaceae (Amagase and Farnsworth, 2011 ▶). Wolf berry has different vernacular names; the most common name, wolfberry, comes from the character“gou”as it is related to the one that means wolf. The name goji is an extrapolation of a number of native words, and it was originally coined in 1973 by researchers at the Tanaduk Botanical Research Institute (TBRI) (Amagaseand Farnsworth, 2011 ▶). Goji plants are native to China, where they grow from the subtropics in the south to the cold, dry climate on Inner Mongolia. Commercial fruit production is concentrated near Inner Mongolia. The fruits are red like a tomato, with a green calyx near the stem. Seeds are small and edible, similar to tomato seeds. Flowers havea purple colour which fades to yellow (Amagase and Farnsworth, 2011 ▶). Geographical origin is one of the most important quality parameters for many foods, as differences in climate, soil and cultivation methods cause differences in the chemical composition of the plants. Ningxia is recognized as the daodi region of goji, increasing market demands pushed the cultivation into new regions in China and gojifields now stretch over different geographical and climatic environments between 82^o^E and 115^o^E, 30^o^N and 45^o^N. These include temperate monsoon climate (Hebei), temperate continental semi-arid climate (Ningxia, Gansu and Inner Mongolia), plateau continental climate (Qinghai), and continental arid climate (Xinjiang) (Li et al., 2017 ▶). These different environmental conditions influence both the appearance and the metabolite profile ofgoji (e.g. amount of polysaccharides, flavonoids, betaine, and carotenoids) (Shen et al., 2016 ▶). Gojiprefers a moderately moist, well-drained soil, but it isalso fairly drought tolerant. The berries are produced and ripen the best in full sun. Wolfberry shrubs have long, arching branches that hold up better with some structural support. The famers can also train gojisonto a trellis, fence or any other solid structure. Due to their vigorous growth habit,goji can be pruned anytime to control its height and shape. Yao et al. (2018) ▶ reported that it does not justify superiority of a specific production area over other areas. Instead, it will be essential to distinguishgoji from different regions based on the specific morphological and chemical traits with the aim to understand what its intended uses are. From an agronomical point of view, each region produces specific cultivars that may differ in chemical composition and biological properties (Wojdylo et al., 2018 ▶). Their undocumented legend, however, is considerably older, as wolf berries are often linked in Chinese lore to ShenNung (Shennong), China^’^s legendary First Emperor, mythical father of agriculture, and herbalist who lived circa 2800 BC. The book was named “Shennong Ben Cao Jing” and supposed to contain all of the emperor^’^s knowledge on the subject of agriculture. There is another important Chinese book written by Li Shi-Zhen in the 16^th^ century that also included important information on the subject of the wolfberry. From a TCM point of view, the nature of wolfberry is calm, and its flavour is sweet. According to TCM theory and practice, wolf berry can act on both the liver channel and the kidney channel, and the major health benefits of wolfberry are its ability to nourish and tonify the liver and kidney (Cieslik and Gebusia, 2012 ▶). It should be noted that wolfberry is used not only as a drug in TCM prescriptions to treat diseases but also as a popular food consumed by Chinese people in their daily life for promotion of general health. According to the regulations of the China State Food and Drug Administration, wolfberry is one of the 87 TCM ingredients that can be used as both normal and functional food (Fiorito et al., 2019 ▶; Shahrajabian et al., 2019 ▶). One theory as to the origin of the wolfberry name stems from speculation that Chinese farmers saw wolves sheltering among the dense wolfberry vines. Most of theworld^’^swolfberry production centres are around areas in North western China, where there are 200,000 acres of farmland dedicated to wolfberry cultivation. Wolfberry plantations can also be found in Inner Mongolia and Shaanxi.Wu et al. (2018) ▶ also reported that northwest regions of China are the main producing area of *L. barbarum*, including Xinjiang, Tibet, Ningxia, Inner Mongolia, Qinghai and Gansu. Wolfberries provide 8 essential amino acids that the body cannot synthesize. One of the most important reasons for the popularity of wolfberryis the fact that they contain a high concentration of an antioxidant called Zeaxanthin. According to various studies, a diet that contains wolfberry can increase a person^’^szeaxanthinlevels by as much as 26. Wolfberry is frequently added to soups, hot pots, and herbal teas, and is also popularly soaked in wines alone or together with other TCM ingredients to make functional wines (Zhang et al., 2015 ▶). On the basis of TCM view, wolfberry is mainly used in treating yin deficiency in the liver and kidney. The dried fruit is commonly used in TCM preparations at a dose of 6-15 g, taken twice or thrice daily (Liu and Tseng, 2005 ▶). Wolfberry can also be a part of a mix of Chinese herbs that is ground to a fine powder and used in honey pills. Nutricosmetics are used for the promotion of skin and hair health. Only angelica and pearl powder are more frequently found in nutricosmetic products in China (Bucheli et al., 2011 ▶). Wolfberry root bark is used for treating inflammation and certain skin diseases. Fresh and dried gojiberryis shown in Figure 1. 

**Medicinal uses and potential health benefits of wolfberry in modern medicine industry**

Wolfberry can provide almost twice vitamin A that a person needs in a day (Liu et al., 2018 ▶). It has almost a third of the daily recommended vitamin C. Moreover, wolfberry is rich in some important and essential minerals including iron and potassium. Endes et al. (2015) ▶ reported that this crop includes essential oils, vitamins (A, A, and C), amino acids, mineral elements (K, P, Ca, Mg, Fe, and Na), and betaine. Wolfberry contains high levels of fibre and protein, which means that itcan help to provide a feeling of fullness without the need to take an inordinate amount of calories. The nutritional and functional properties of wolfberryare provided by a rich variety of components, including amino acids, polyphenols, carotenoids, polysaccharides, organic acids and their derivatives (Bertoldi et al., 2019 ▶; Zhao et al., 2019 ▶). Mocan et al. (2018) ▶ reported that wolfberry isa rich source of bioactive compounds with functional properties that need further risk/benefit evaluation when used in foods or health promoting formulations. There are many varieties of wolfberry grown in differentparts of the world. As the popularity of the berry continues to grow, more and more varieties will likely appear as the vine is cultivated for commercial purposes. But it is said that the most powerful and nutrient rich wolfberryin China still comes from the vines of Himalayan valleys. Goji is a good source of fibre, protein, carotenoids, and polysaccharides (Shahrajabian et al., 2018 ▶). It also has a lot of biological activities, including antidiabetes, antiproliferative activity, preserving retinal function, and antioxidant activity (Song and Xu, 2013 ▶; Magiera and Zareba, 2015 ▶; Zhang et al., 2016 ▶). Donno et al. (2015) ▶ mentioned that wolfberry is as a rich source of antioxidant compounds with health promoting properties comparable with other common fruit species. Recent studies have shown that antioxidant activities of some natural products are correlated with defence against oxidative stress and different human diseases including cancer, arteriosclerosis and aging process (Willcox et al., 2004 ▶). Compounds of nutritional value in gojiare diverse and theyincludepolysaccharides, carotenoids, polyphenols, essential oils, betaine, vitamins, amino acids and oligo elements (Forino et al., 2016 ▶). Wolfberry is rich in sugars and lipids (Blasi et al., 2017 ▶). Wojdylo et al. (2018) ▶ indicated that apart from being natural, nontoxic colorants in drinks and cosmetics, gojicarotenoids show biological activity (e.g. they act as antioxidants or precursors of vitamin A). Xie et al. (2016) ▶ reported that *LyciumBarbarum* can be utilized as a pharmaceutical for treatment and as an ingredient in Chinese cooking. Cheng et al. (2015) ▶ reported that wolfberry has long been used to promote fertility and as a potent anti-aging and antioxidant agent. Wolfberry also has fatty acids (hexadecanoic acid, linoleic acid and myristic acid) (Blasi et al., 2017 ▶), and amino acids (proline, betaine and taurine) (Potterat, 2010 ▶). Yan et al. (2014) ▶ in their experiment, indicated that the contents of nutritional components in the different tissues were significantly different. The pollen and the fruit contained highly unsaturated fatty acids. All the tissues were good sources of mineral elements, polysaccharides and phenolic compounds. Furthermore, they have found that Ningxia wolfberry pollen, leaf and flower can be a potential source of nutrients for humans and animals.It has also effectiveness in aging, increased metabolism, immune system, liver function and glycemic control (Silva et al., 2017 ▶). However, their benefits are attributed to the bioactive component polysaccharide-protein complex 4 (LBP4), which is composed of six monosaccharides (galactose, glucose, rhamnose, arabinose, and mannoseandxylose) (Lu and Zhao, 2010 ▶; Carnes et al., 2013 ▶). Soaresde Sousa et al. (2016) ▶ noted that gojiis rich in vitamins and minerals that protect the central nervous system, reduces the risk of glaucoma and has antitumor activity, prevents chronic diseases such as hypercholesterolemia, diabetes, hepatitis, and helps in reducing fatigue and greater resistance in exercise, being a strong ally in the prevention of aging. It has been found that the flavonoids from wolfberryprotect the blood cells and mitochondria against oxidative damages (Luo et al., 2004 ▶). Jin et al. (2013) ▶ demonstrated that *L. barbarum *polysaccharides have various important biological activities, such as antioxidant, immunomodulation, antitumor, neuroprotection, radioprotection, anti-diabetes, hepatoprotection, anti-osteoporosis and anti-fatigue. Masci et al. (2018) ▶ also concluded that the purified components of wolfberrymay be potentially useful as adjuvants in the treatment of diabetes and its correlated illnesses. A human supplementation trial showed that daily intake of wolfberryincreased plasma levels of zeaxanthin (Hempel et al., 2017 ▶). Some health benefits of wolfberry are boosted immune system and flu protection, potential weight loss aid, antioxidants for eyes and skin, maintain blood sugar, increased testosterone, restored body homeostasis and strengthened body energy (Protti et al., 2017 ▶). Some researchers reported that the carotenoid profile of wolfberryis the subject of different reports, where zeaxanthin-dipalmitatewas confirmedas the major carotenoid of wolfberry (Hempel et al., 2017 ▶; Fratianni et al., 2018 ▶). Fratianni et al. (2018) ▶ mentioned that the dried samples of wolfberrycould be used as a dietary source of carotenoid and be worthy of development and utilization. Dried fruits can be eaten raw and used in confectionary or in bakery products, added to trail mix, cereals, muffins, energy bars or soups. Wolfberrycontains not only high amounts of antioxidants, carotenoids, vitamin A and zeaxanthin, but also it is rich in vitamins B and C and polysaccharides (Skenderidis et al., 2018 ▶; Senica et al., 2018 ▶). In addition, flavonoids such as rutin, gentistic acid and quercetin are the main active compounds present in the leaves of *Lycium barbarum* (Dermesonlouoglou et al., 2018 ▶). Goji extracts were proven to possess biological activities, e.g. anti-ageing effects, increased metabolism, antioxidant properties, anti-diabetes and glucose control, immunomodulation, anti-glaucoma, neuroprotection, anti-fatigue/endurance, cytoprotection and antitumour activity (Potterat, 2010 ▶). Numerous studies indicated the powerful antioxidant potentialities achieved from *L. barbarum *molecules, with various health protective effects (Abdennacer et al., 2015 ▶). It waswell documented that several traditional herb and plant extracts have antioxidant properties and are potential candidates for the prevention and treatment of reactive oxygen species (ROS)-induced diseases (reactive oxygen species) (Leontopoulos et al., 2017 ▶). Driedgojifruits (*L. Chinese*) havethe highest content of total polyphenols and vitamin C based on the cellular juice concentration due to fruits dehydration. The DPPH (2, 2-diphenyl-1-picryl-hydrazyl-hydrate) method was affected by the content of vitamin C(Rocchetti et al., 2018 ▶). Macronutrients include carbohydrates, protein, fat, and dietary fibre. It was shown that 68% of the mass of dried wolfberryexists as carbohydrate, 12% as protein, and 10% each as fibre and fat each, giving a total caloric value of 370 (kilo) calories in a 100 gram serving, of which 272 come from carbohydrates and 90 from fat. Yu et al. (2006) ▶ mentioned that the pharmacological activities associated with *L. barbarum* include hypoglycemia, immunomodulation, anti-hypertension, lipotropic, protecting hepatic function, anti-aging, anti-fatigue, antioxidant, etc. Some researches indicated that components of berry fruits especially wolfberry may inhibit replication oftheinfluenzavirus both directly and indirectly, e.g. by blocking surface flycoproteins of influenza virus and stimulating the immune system of the organism; Inconsequence to its properties berry fruits, wolfberry included, are raw materials of potential use in the prevention and treatment of influenza (Gramza-Michalowska et al., 2017 ▶).Some chemical compounds of goji berriesarepresented in Table 1. Nutritional composition of fresh and dried berries (g/100 g) is shown in Table 2. Average values of mineral elements in fresh and dried goji berries (mg/100 g) arepresented in Table 3. The most important health benefits of goji berries are shown in Table 4. 

**Table 1:** Some chemical compounds of gojiberry (Ma et al., 2019)

Composition	
Moisture (%)	10.3
Crude protein (%)	8.9
Crude oil (%)	4.1
Fiber (%)	7.3
Total phenol (mg GAE/100 mL)	3.4
Antioxidant acid (%)	20.8
Myristic acid (%)	0.1
Stearic acid (%)	2.9
Palmitic acid (%)	8.2
Arachidic acid (%)	1.8
Oleic acid (%)	21.7

**Table 2:** Nutritional composition of fresh and dried berries (g/100 g) (mean±standard deviation) (Niro et al., 2017)

	Moisture	Fats	Proteins	Carbohydrates	Insoluble Fibre	Total	Ash
Fresh	77.4±0.4	1.1±0.02	2.5±0.12	15.3	2.2±0.02	2.9	0.84±0.11
Dried	9.3±0.02	4.4±0.45	10.2±0.22	61.3	8.8±0.01	11.4	3.4±0.16

**Table 3:** Average values of mineral elements in fresh and dried goji berries (mg/100 g) (mean±standard deviation) (Niro et al., 2017 ▶)

	Fresh	Dried
Ca	26.6±4.90	101.3±22.60
K	276.2±41.00	881.9±239.70
Mg	12.7±2.80	45.9±9.20
Na	57.3±8.70	209.8±72.30
P	48.4±9.26	174.3±32.10
Co	0.001	0.001
Cu	0.3±0.04	0.8±0.25
Fe	0.9±0.22	3.4±1.57
Mn	0.2±0.03	0.5±0.18
Zn	0.5±0.12	1.5±0.62
Se (µg/100g)	0.1±0.01	017±0.03
Mo(µg/100g)	0.00	0.00

**Table 4:** The most important health benefits of gojiberry

Very nutritious
Excellent source of antioxidants
Anti-aging benefits
Prevent cancer growth
Improve blood sugar control
Boost energy levels
Help to lose weight
Improve cholesterol levels
Boost the immune system

## Discussion

Traditional Chinese Medicine (TCM) has been used for thousands of years by different generations in China and other Asian countries as foods to promote good health and as drugs to treat diseases. It has been widely used in Asian countries such as China, Japan, Korea, Vietnam, and Thailand for many years. Wolfberry is widely distributed in the arid and semi-arid regions of China, Japan, Korea, Europe, North America and the Mediterranean. The northwest regions of China are main producing areas of *L. barbarum*, including Xinjiang, Tibet, Ningxia, Inner Mongolia, Qinghai and Gansu.

Currently, China is the major supplier of *L. barbaru* in the world. Wolfberry, as a Chinese traditional herb and food supplement, contains many nutrients and phytochemicals, such as polysaccharides, scopoletin, the glucosylated precursor, amino acids, flaconoids, carotenoids, vitamins and minerals. It has anticancer, antioxidant, retinal function preservation, anti-diabetes, immune function boosting and anti-fatigueproperties. Widely used in TCM, wolf-berrycan be sold as a dietary supplement or classified as nutraceutical food due to itslong and safe traditional use. Modern goji enhances the body’s ability to adapt to a variety of noxious stimuli; it significantly inhibits the generation and spread of cancer cells and can improve eyesight and increase reserves of muscle glycogen and liver glycogen which may increase human energy and has anti-fatigue effects. Wolfberrymay improve brain function and enhance learning and memory. It may boost the body^’^s adaptive defences, and significantly reduce the levels of serum cholesterol and triglyceride; it may help weight loss and obesity and treat chronic hepatitis and cirrhosis. Today, it is considered as functional food with many beneficial effects, which is why they have become more popular recently, especially in Europe, North America and Australia, wherethey are considered superfood with highly nutritive and antioxidant properties. Geographical origin of wolfberryisone of the most important quality parameters in TCM since the differences in climate, soil, and cultivation methods cause differences in the chemical composition of the plants (Soleymani and Shahrajabian, 2011 ▶; Soleymani and Shahrajabian, 2012 ▶; Shahrajabian and Soleymani, 2017 ▶; Soleymani et al., 2010 ▶; Shahrajabianet al., 2011 ▶; Soleymani et al., 2011 ▶; Soleymani et al., 2012 ▶; Shahrajabian et al., 2013 ▶; Soleymani et al., 2016 ▶; Soleymani and Shahrajabian, 2017 ▶; Shahrajabian et al., 2017 ▶). 

Now, gojiis enjoying the enormous popularity worldwide and it is madeas tea, beer, cookies, cuttings, dessert, drinks, eye cream, extract, powder, essential oil, facial cream, face mask, jelly, smoothie, jam, muffin, supplements, etc. Although TCM in China is partly integrating with western medicine science, researchers shall learn more from TCM and carry out more studies and researchers in order to explore the advantages of TCM in daily life. All in all, this review article allowed verifying that wolfberry as a source of compounds with valuable nutritional and bioactive properties;wolfberrycould be useful for incorporation into foods with functional properties. Wolfberry has huge health benefits that attract international markets. Wolfberry which is known as the super fruit and super food in TCM for the claimed health benefits and it should be part of daily diet. 
